# Beyond Me, Too

**DOI:** 10.1200/JGO.2016.005116

**Published:** 2016-05-18

**Authors:** Richa Vaish, Harsh Dhar, Anil K. D’Cruz

**Affiliations:** Tata Memorial Hospital, Mumbai, India

## To the Editor:

The article by Gyawali^1^ highlights the important issue of rising costs associated with the cancer treatment that is important to our part of the world. The article rightly enumerates challenges faced by the health care system in low- and low-middle–income countries (LLMICs). Increasing financial burden associated with cancer treatment is a cause of concern not only in developing countries, but in the developed world as well. The author has highlighted the current paradox that plagues the medical fraternity. On one hand, we discuss breakthrough research at premier scientific meetings (eg, those of the American Society of Clinical Oncology), while on the other, the majority of the world is unable to provide basic cancer care for patients.^1^

The author has correctly identified LLMICs as being fertile ground for conducting trials. It is surprising, however, that Gyawali limits this to the conduct of phase I and II trials only. A large patient pool, low overhead costs, and trained manpower make a compelling case for the conduct of phase III trials in these countries.^2,3^ It is a well-known fact that more than half of phase III trials sponsored by US-based pharmaceutical companies are conducted outside the United States—a large number of them in LLMICs.^4^ Such an approach seems attractive because it should reduce the cost of research and drug development, with a resultant impact on entry pricing of new treatments. There will also be an added ethical responsibility among drug developers to make the new molecule available in countries where the initial research was conducted.

High-impact research from the developing world has received global attention in the recent past.^5,6^ Importantly, all of these studies have low-cost innovations at the core of their design with results that have global applicability^7^ and implications for health expenditures, which is a concern in developed countries as well.^8^ This makes a case in point for research to focus on cost-effective innovations rather than high-end “me too” molecules only. Exploiting the unique biologic characteristics of cancer with simple approaches such as drug repurposing, metronomic chemotherapy, and using the critical perioperative period to improve long-term cancer outcomes are other attractive, cost-effective options.^9-11^

With the increasing burden of cancer, a large proportion of health expenditures is on its treatment. The ability to deliver high-quality, affordable cancer care with its increasing cost is a challenge in developed countries as well.^8^ Practice-changing, well-conducted randomized controlled trials need not focus only on high-end, costly targeted molecules. The need of the hour is to incorporate cost-effectiveness analysis in research toward the goal of equitable cancer care for all.
